# Prevalence and diversity of antifungal resistance in *Fusarium* isolates across clinical and agricultural settings in the United States

**DOI:** 10.1128/aac.01208-25

**Published:** 2025-11-18

**Authors:** Emilie Applebach, Kincer Amburgey-Crovetti, Wayne L. Bakotic, Tika B. Adhikari, Nathan P. Wiederhold, Shelby Hodges, Tomoko Y. Steen, Richard Calderone, Dongmei Li

**Affiliations:** 1 Department of Microbiology and Immunology, Georgetown University Medical Center 12231, Washington, DC, USA; 2 Bako Diagnostics 480715 , Alpharetta, Georgia, USA; 3 Department of Entomology and Plant Pathology, North Carolina State University 6798 , Raleigh, North Carolina, USA; 4 Fungus Testing Laboratory, Department of Pathology and Laboratory Medicine, University of Texas Health Science Center at San Antonio 14742, San Antonio, Texas, USA; University of Iowa, Iowa City, Iowa, USA

**Keywords:** *Fusarium *species complexes, azole drug resistance, multiple drug resistance, intrinsic resistance, resistance evolution

## Abstract

Disseminated fusariosis and chronic superficial fusariosis require effective strategies to control fungal growth and prevent systemic spread. This study presents an updated, comprehensive analysis of antifungal susceptibility in 174 *Fusarium* isolates collected from clinical and agricultural sources across 26 US states. Using a standardized panel of 14 antifungal agents, we compared resistance profiles across isolates to identify key epidemiological and resistance trends. Among clinical isolates, over 60% exhibited minimum inhibitory concentrations ≥ 32 µg/mL to all four clinical azoles, 49% to amphotericin B, and 74% to 5-flucytosine. Notably, nearly half of the azole-resistant clinical isolates also showed high-level resistance (≥32 µg/mL) to all five agricultural azoles. Our data demonstrate the complex, multifactorial nature of antifungal resistance across *Fusarium* species complexes (SCs), environmental sources, and drug classes. The widespread use of agricultural azoles may be a contributing factor to the observed prevalence of resistant *Fusarium oxysporum* and *Fusarium solani* SCs, as suggested by correlations between voriconazole and oxiconazole resistance, though causality cannot be established from this study. Resistance in *Fusarium fujikuroi* SC may emerge primarily in clinical settings, with potential links to agricultural azole exposure remaining uncertain. Despite high resistance to individual antifungal classes, the relatively low incidence of multidrug and pan-drug resistance, observed in fewer than 30% of clinical strains across azoles and other antifungal agents, suggests that combination therapy may still offer clinical benefit. These findings highlight the urgent need for continued antifungal resistance surveillance and the development of integrated, evidence-based strategies for the effective management of *Fusarium* infections.

## INTRODUCTION

Global ecological changes are reshaping the landscape of fungal diseases and influencing antifungal drug susceptibility (1). The increasing incidence of fungal infections originating from the environment has prompted the rise of the “One Health” movement—an integrated approach that brings together human, animal, and environmental health sectors both in the United States and globally. Our initial aims are to raise awareness and develop effective tools to improve disease control and management. Among the environmental fungi of concern, *Aspergillus* species and *Fusarium* spp. stand out as prominent trans-kingdom pathogens, capable of infecting both humans and plants (2–4).

*Fusarium* infections exhibit a distinct clinical profile compared to those caused by *Aspergillus* species. While *Aspergillus* primarily induces systemic infections via the respiratory tract in humans, *Fusarium* infections more commonly involve superficial sites, typically following local trauma or wounds in immunocompetent individuals (5, 6), such as combat-related injuries (7–9). In civilian populations, however, keratitis and onychomycosis are the most common clinical manifestations (10–14). These infections often respond poorly to current treatment strategies, leading to prolonged disease courses, chronic inflammation, and, in some cases, permanent vision loss or disfigurement (15, 16). This site-specific tropism may be related to biological characteristics of *Fusarium*, including its high oxygen requirement and preference for temperatures closer to those of the external environment (17).

Although invasive cases are relatively rare, *Fusarium* can cause invasive fusariosis in immunocompromised individuals, with mortality rates frequently exceeding 50% despite antifungal therapy (18–21). Similar to invasive aspergillosis, the respiratory tract is the primary portal of entry; however, breaches in the skin barrier represent a secondary route of infection (22). This highlights the potential for uncontrolled fungal spread from superficial colonization to deep tissue invasion. As immune function declines with age or comorbidities, the risk of progression from localized to systemic *Fusarium* infection may increase. Notably, animal models have demonstrated that *Fusarium* spores can persist in host tissues in a latent state and later reactivate (23).

The combination of therapeutic resistance and aggressive clinical progression underscores an urgent need for more effective treatment strategies. Currently, five major classes of antifungal agents are used in clinical practice: azoles, polyenes, echinocandins, allylamines, and flucytosine. Azoles, including fluconazole (FCZ), voriconazole (VOZ), and posaconazole (POZ), inhibit ergosterol synthesis by targeting the 14α-lanosterol demethylase enzyme. Polyenes, such as amphotericin B (AmB), bind to ergosterol in the fungal membrane, causing membrane disruption and cell death. Echinocandins, like caspofungin and micafungin (MIF), inhibit β-1,3-glucan synthase, weakening the fungal cell wall; however, their activity against *Fusarium* is limited. Allylamines, such as terbinafine (TER), interfere with an earlier step in ergosterol synthesis by inhibiting squalene epoxidase. 5-flucytosine (5-FC), a pyrimidine analog that inhibits fungal DNA and RNA synthesis, is not commonly used against *Fusarium* due to its toxicity profile and limited efficacy. Despite the availability of these drug classes, treatment outcomes for *Fusarium* infections remain suboptimal (24), and clinical breakpoints have yet to be established for each form of infection.

Despite their limited efficacy, second-generation azoles, such as VOZ and POZ, along with AmB, remain the primary treatment options for *Fusarium* infections in the absence of more effective alternatives (24). The rising prevalence of antifungal resistance among *Fusarium* isolates (25) presents two major challenges: it narrows therapeutic options for invasive infections in immunocompromised patients and complicates the management of chronic superficial infections, which often require prolonged treatment. In an analysis of breakthrough invasive fungal infections in patients receiving VOZ or POZ prophylaxis, *Fusarium* accounted for 9% of cases. Notably, invasive fusariosis was more frequently associated with POZ-related breakthrough infections, whereas VOZ prophylaxis was more commonly linked to breakthrough mucormycosis (26).

Environmental factors, particularly in agriculture, may contribute to the drug resistance observed in clinical *Fusarium* isolates (27). Azole-based fungicides have been used extensively in agriculture for decades to prevent crop loss and reduce mycotoxin contamination (28, 29), long before their widespread adoption in clinical settings. This overlap in usage has been implicated in the emergence of azole cross-resistance, particularly in *A. fumigatus* (30)*,* where environmental exposure to triazoles has been linked to well-characterized resistance mutations such as TR34/L98R in the *CYP51A* gene (31–34). Since both clinical and agricultural azoles target the same fungal ergosterol biosynthesis pathway, it is plausible that similar selective pressures may contribute to resistance in *Fusarium* species as well.

As a common soil-borne pathogen of many agricultural crops, *Fusarium* is routinely exposed to triazole fungicides. These plant-protective azoles are inexpensive, produced in large quantities, and are environmentally persistent. Toda et al. (28) reported a fourfold increase in global triazole usage between 2006 and 2016, reaching 2.9 million kilograms annually. However, unlike *A. fumigatus,* genetic resistance markers associated with triazole exposure are rarely identified in clinical *Fusarium* isolates. To date, only one study from Malaysia has reported a 23-base pair deletion in the *CYP51A* promoter region of several *Fusarium solani* strains resistant to VOZ (35). *O*verall, the molecular mechanisms underlying azole resistance in *Fusarium* remain poorly understood. Future investigations using our clinical isolates may uncover novel genetic markers and resistance pathways relevant to triazole exposure.

To identify potential genetic markers of antifungal resistance, we conducted comprehensive susceptibility profiling of over 100 clinical *Fusarium* isolates and 42 agricultural strains from the United States. This included cross-testing their susceptibility to both clinical and agricultural azoles, as well as representative compounds from four additional major antifungal classes. This work provides a foundational data set to support future investigations into resistance mechanisms and species-specific genetic variation among clinical isolates. By establishing resistance patterns across a diverse panel of strains, our study aims to clarify the relationship between antifungal resistance and potential environmental contributions. Ultimately, these findings will inform the development of targeted diagnostics and more effective treatment strategies for *Fusarium* infections.

## RESULTS

### Predominance of *F. solani* species complex among clinical isolates across 26 States in the United States

**Fig 1:**
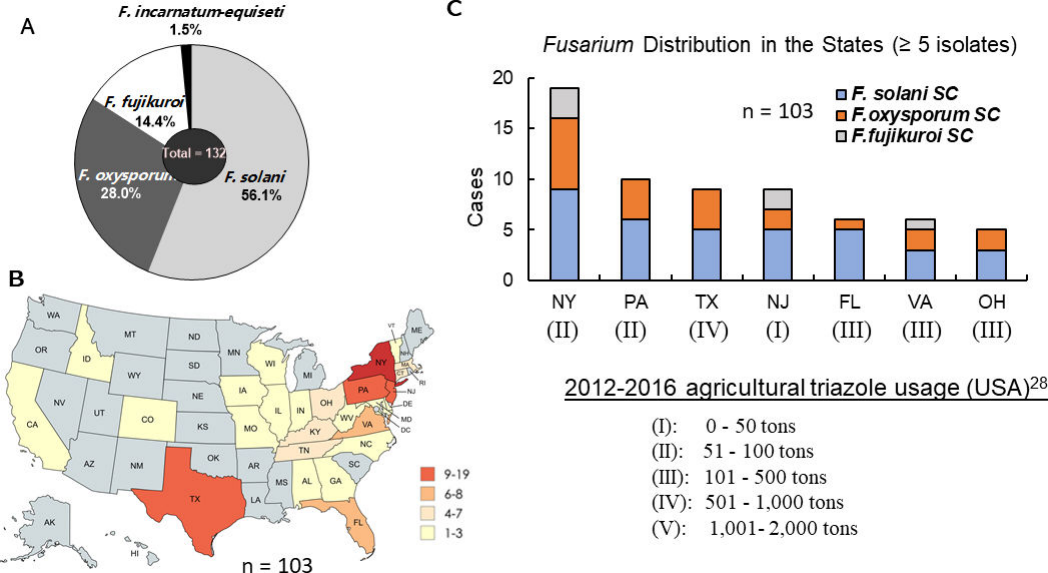
Species distribution of clinical *Fusarium* isolates in the United States. (**A**) Overall species distribution of 132 clinical isolates, representing four SCs: *F. solani* SC*, F. oxysporum* SC, *F. fujikuroi* SC, and F. *incarnatum-equiseti* SC. The first three species follow a 4:2:1 ratio pattern. (**B**) Geographic distribution of 103 toenail isolates collected across 26 states. Created using MapChart. (**C**) Species distribution in the seven states with more than five isolates. Roman numerals below state abbreviations indicate the average agricultural triazole usage from 2012 to 2016 (28), ranging from low (I) to high (V).

A total of 132 clinical *Fusarium* isolates were included to assess their overall antifungal resistance in the study. These isolates were collected from 26 states across the United States and were grouped into three sets (Set A, Set B, and Set C) based on strain collection scenarios (Table 1). Among them, 74 isolates (56.1%) belonged to the *F. solani* species complex (SC), 37 (28.0%) to the *F. oxysporum* SC, 19 (14.4%) to the *F. fujikuroi* SC, and 2 (1.5%) to the *F. incarnatum-equiseti* SC (Fig. 1A).

**TABLE 1:** *Fusarium* isolates (*n* = 175) from clinical and field settings in the United States

Set serial	Name	species (species complex)	Source (location)	Anatomic location
Set A	A1–A17	*F. solani* (FSSC)	CDC	Unknown (ukn)^*b*^
Set B	B1–B12	*F. solani* (FSSC): B3, B6, B7, B9	MI, NJ, CA, MA	foot, tissue,thigh, nose
		*F. oxysporum* (FOSC): B8	CA	tongue
		F. fujikuroi (FFSC): B1, B2, B4,	FL, ukn, MA	BAL, nail, cornea
		B10, B11,B12	MA, MI, ukn	BAL, BAL, foot
		F.incarnatum-equiseti(FIESC):B5	ukn	BAL
Set C	R01–R104	*F. solani* (FSSC): 53 isolates	18 states	Infected human nails Across 26 states^*a*^
		*F. oxysporum* (FOSC): 36 isolates	13 states	
		*F. fujikuroi* (FFSC): 14 isolates	10 states	
		*F. incarnatum-equiseti* (FIESC): 1 isolate	MA	
Set D	K01–K37	*F. oxysporum* (FOSC): 37 isolates	NC	Infected tomato plant
	K38–42	*F. oxysporum* (FOSC): 5 isolates	MD	Farm field, garden

^*a*^ The 26 states are Alabama (AL), California (CA), Colorado (CO), Connecticut (CT), District of Columbia (DC), Delaware (DE), Florida (FL), Georgia (GA), Idaho (ID), Illinois (IL), Indiana (IN), Iowa (IA), Kentucky (KY), Maryland (MD), Massachusetts (MA), Missouri (MO), New Jersey (NJ), New York (NY), North Carolina (NC), Ohio (OH), Pennsylvania (PA), Tennessee (TN), Texas (TX), Vermont (VT), Virginia (VA) and West Virginia (WV).

^*b*^ Ukn: unknown collection site.

While geographic data were unavailable for Set A and incomplete for some strains in Set B, we focused on toenail-derived isolates from Set C (*n* = 103) to map *Fusarium* distribution across the 26 states/regions (Fig. 1B). Due to the chronic nature of toenail infections and the potential for prolonged exposure to clinical azoles, these isolates provide an ideal cohort for analyzing resistance patterns to both clinical and agricultural azoles. The toenail isolates showed variable species distribution across the 26 states. As depicted in Fig. 1B, New York had the highest number of isolates (19), followed by Pennsylvania with 10 isolates, and Texas and New Jersey with 9 isolates each. Among the 19 isolates from New York, 9 from New Jersey, and 6 from Virginia, *F. solani* SC/*F. oxysporum* SC/*F. fujikuroi* SC accounted for 9/7/3, 5/2/2, and 4/2/1, respectively (Fig. 1C), which is roughly consistent with the overall 4/2/1 ratio of those three species in Fig. 1A. However, in other states with more than five strains, including Texas, Pennsylvania, Florida, and Ohio, *F. solani* SC and *F. oxysporum* SC were predominant, with no isolates of *F. fujikuroi* SC.

### Preliminary associations between *Fusarium* SC distribution and agricultural azole usage

Interestingly, when comparing species distribution to agricultural azole usage data in the United States from 2012 to 2016 (28), the three states with a close 4/2/1 ratio of *F. solani* SC/*F. oxysporum* SC/*F. fujikuroi* SC (NY, NJ, and VA) were located in regions reporting lower agricultural azole usage (0–500 tons; categories I–III in Fig. 1C). In contrast, states where *F. fujikuroi* SC was absent and *F. oxysporum* SC increased (e.g., Pennsylvania and Texas), or where *F. solani* SC predominated (Florida), were among regions with higher agricultural azole usage (51–1,000 tons; categories II–IV). These patterns raise the hypothesis that agricultural azole exposure may influence *Fusarium* species distributions. However, this interpretation should be considered speculative, as other ecological and environmental factors are also likely to contribute, and our limited sampling does not allow for definitive conclusions. More extensive, stratified environmental sampling will be needed to test this hypothesis rigorously.

### Widespread distribution of azole-resistant *Fusarium*

Epidemiological cutoff values (ECVs) represent the highest minimum inhibitory concentration (MIC) expected for organisms without detectable resistance mechanisms and are commonly used to distinguish wild-type isolates from those with acquired or mutational resistance. ECVs are particularly useful when clinical breakpoints or interpretive MIC values have not been established for a specific organism, as is the case for *Fusarium* species.

Since only two *F. incarnatum-equiseti* SC isolates were present in our collection, their antifungal susceptibility data were excluded from further resistance analysis. The remaining 130 clinical isolates—including 74 *F*. *solani* SC, 37 *F*. *oxysporum* SC, and 19 *F*. *fujikuroi* SC—exhibited widespread reduced susceptibility to commonly used clinical azoles, with geometric mean (GM) EC values often exceeding the established ECVs (36). Among the tested azoles, VOZ consistently showed the lowest GM values: 16.76 µg/mL for *F. solani* SC, 11.42 µg/mL for *F. oxysporum* SC, and 13.83 µg/mL for *F. fujikuroi* SC (Table 2). Notably, while the GM values for *F. solani* SC and *F. oxysporum* SC fell below their respective ECVs, strains of *F. fujikuroi* SC exhibited ECVs higher than the previously reported values (36). The drugs with GM values approaching the ECV threshold of 32 µg/mL included FCZ and itraconazole (ITZ) in the *Fusarium solani* SC, with GM values of 32.3 µg/mL and 34.81 µg/mL, respectively. Notably, all tested strains demonstrated poor susceptibility to POZ, with GM values ranging from 41.31 µg/mL in *F. fujikuroi* SC to 64 µg/mL in *F. oxysporum* SC.

**TABLE 2:** Reduced susceptibility of clinical isolates to clinical and agricultural drugs**^*e*^**

			*F. solani* SC (*n* = 74)	*F. oxysporum* SC (*n* = 37)	*F. fujikuroi* SC (*n* = 19)
Clinical azoles	VOZ	MIC_50_**^*a*^**/MIC_90_**^*a*^**	32/64	32/64	64/64
		GM**^*b*^**	16.76 [32]**^*c*^**	11.42 [16]	13.83 [4]
		Range	0.125–64	0.125–64	0.125–64
		Mode**^*d*^**	64	64	64
	POZ	MIC_50_/MIC_90_	64/64	64/64	64/64
		GM	52.6 [32]	64 [8]	41.31 [2]
		Range	1–64	64–64	1–64
		Mode	64	64	64
	ITZ	MIC_50_/MIC_90_	64/64	64/64	64/64
		GM	34.81 [32]	50.16 [32]	41.31 [32]
		Range	0.125–64	1–64	0.125–64
		Mode	64	64	64
	FCZ	MIC_50_/MIC_90_	64/64	64/64	32/64
		GM	32.30 [32]	33.22 [16]	42.85 [4]
		Range	0.125–64	0.125–64	2–64
		Mode	64	64	64
	CLZ	MIC_50_/ MIC_90_	64/64	32/64	64/64
		GM	36.14	51.12	44.43
		Range	0.125–64	2–64	8–64
		Mode	64	64	64
Field (agricultural) azoles	TRI	MIC_50_/ MIC_90_	64/64	64/64	64/64
		GM	53.57	59.37	55.09
		Range	1–64	16–64	4–64
		Mode	64	64	64
	FLU	MIC_50_/MIC_90_	64/64	64/64	64/64
		GM	39.69	42.38	24.79
		Range	0.125–64	0.5–64	0.125–64
		Mode	64	64	64
	DIN	MIC_50_/MIC_90_	64/64	64/64	64/64
		GM	53.57	47.42	21.42
		Range	0.5–64	0.25–64	0.125–64
		Mode	64	64	64
	OXI	MIC_50_/ MIC_90_	64/64	64/64	64/64
		GM	36.48	22.84	20.66
		Range	0.125–64	0.25–64	0.25–64
		Mode	64	64	64
	EPO	MIC_50_/ MIC_90_	64/64	64/64	16/64
		GM	44.42	30.25	14.34
		Range	0.125–64	1–64	0.125–64
		Mode	64	64	64
Non-azoles	AmB	MIC_50_/ MIC_90_	16/32	32/64	16/32
		GM	10.3 [8]	18.94 [8]	13.33 [4]
		Range	0.125–64	0.125–64	0.25–64
		Mode	64	64	64
	5-FC	MIC_50_/ MIC_90_	32/64	64/64	32/64
		GM	14.43	17.57	24.79
		Range	0.125–64	0.25–64	2–64
		Mode	64	64	64
	MIF	MIC_50_/MIC_90_	64/64	64/64	64/64
		GM	36.84	45.68	34.42
		Range	0.125–64	16–64	0.125–64
		Mode	64	64	64
	TER	MIC_50_/MIC_90_	64/64	64/64	16/64
		GM	16.15	31.41	7.17
		Range	0.125–64	0.125–64	0.125–64
		Model	64	64	64

^*a*^ Drug concentrations required to inhibit 50% (MIC₅₀) or 90% (MIC₉₀) of tested strains, representing population-level inhibition.

^*b*^ Refers to geometric means.

^*c*^ ECV (epidemiological cutoff value) is based on those reported in reference (36); otherwise, a cutoff of 32 µg/mL is used.

^*d*^ Refer to the predominant MIC value in each group.

^*e*^ VOZ, voriconazole; POZ, posaconazole; ITZ, itraconazole; FCZ, fluconazole; CLZ, clotrimazole; TRI, triflumizole; FLU, flutriafol; DIN, diniconazole; OXI, oxiconazole; and EPO: epoxiconazole, AmB, amphotericin B; 5-FC, 5-flucytosine; MIF, micafungin; TER, terbinafine.

Compared to clinical azoles, reduced susceptibility to agricultural azoles was even more pronounced, especially among clinical *F. solani* SC and *F. oxysporum* SC strains. In *F. solani* SC group, the GM values for all five agricultural azoles exceeded 36.48 µg/mL, while in *F. oxysporum* SC, they were comparable to or slightly higher than those for clinical azoles (Table 2). Among the three groups, *F. fujikuroi* SC showed relatively lower GM values to epoxiconazole (EPO, 14.34 µg/mL), oxiconazole (OXI, 20.66 µg/mL), and diniconazole (DIN, 21.42 µg/mL). Nevertheless, the GM MICs for the first-line antifungals VOZ and POZ were high among the tested isolates.

### Cross-resistance to clinical and agricultural azoles in *F. oxysporum* SC and *F. solani SC*

**Fig 2:**
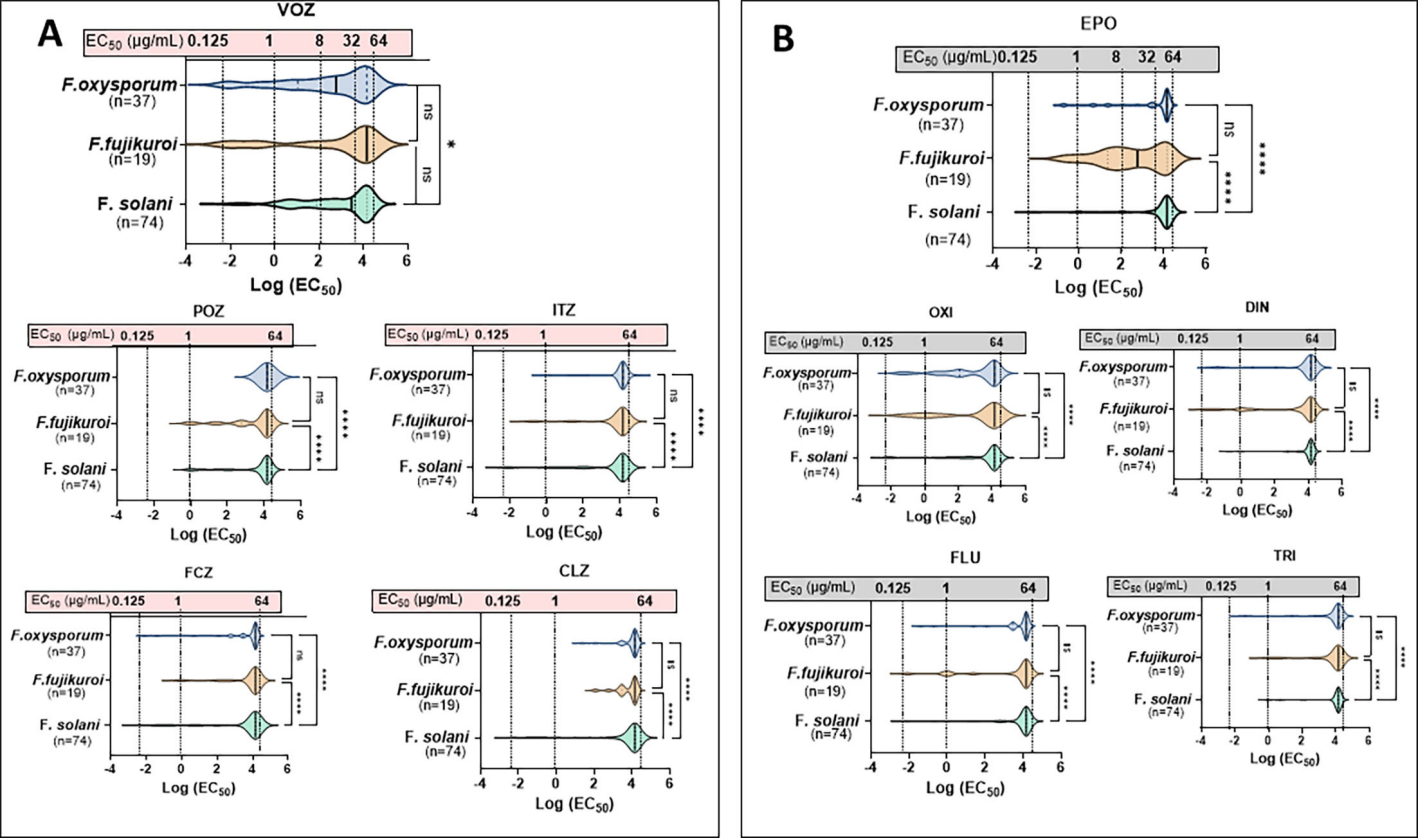
The overall more resistant clinical isolates to agricultural (field triazoles) than clinical azoles. *F. fujikuroi* SC demonstrated significantly less resistance to agricultural azoles, whereas *F. solani* SC exhibited higher resistance to field azoles. Violin plot of EC_50_ distribution for 130 clinical *Fusarium* isolates in three major SCs against clinical (**A**) and agricultural (**B**) azoles. The data set includes 74 isolates of *F. solani* SC, 37 isolates of *F. oxysporum* SC, and 19 isolates of *F. fujikuroi* SC. The x-axis is presented on a natural logarithm (LN) scale, with corresponding EC_50_ values (µg/mL) overlaid on each graph and connected by a dotted line. A natural logarithm (LN) value of 0 corresponds to an EC_50_ of 1. The solid black line within each violin plot represents the median EC_50_ for each group. Clinical azoles include VOZ, POZ, ITZ, FCZ, and clotrimazole (CLZ). Agricultural azoles compose TRI (triflumizole), FLU (flutriafol), DIN, OXI, and EPO. Medians between groups were statistically analyzed using one-way ANOVA, followed by the Kruskal-Wallis test. Significance levels are indicated as follows: *P < 0.05, **P < 0.01, ***P < 0.001, and ****P < 0.0001.

As most of our collected isolates showed no growth inhibition (e.g., EC_90-–100_) at the highest tested concentration (64 µg/mL), particularly for azoles, we used the EC_50_ (50% growth inhibition) to evaluate antifungal susceptibility in each *Fusarium* strain. The EC_50_ values for each azole are spread on a log scale in violin graphs to display resistance distribution against each SC group (Fig. 2), where a log scale of “4” represents an EC_50_ of 64 µg/mL and “0” represents 1 µg/mL. The broader rightward shift in the graphs indicates a higher proportion of resistant strains, whereas a long leftward tail reflects a susceptible population within each species SC.

The EC_50_ distributions for clinical azoles (VOZ, POZ, ITZ, FCZ, and CLZ) against 130 isolates are shown in Fig. 2A, while those for agricultural azoles (EPO, OXI, DIN, FLU, and TRI) are shown in Fig. 2B. Among the clinical azoles, VOZ exhibited the strongest inhibitory activity across all three SCs. Notably, *F. oxysporum* SC showed a significantly lower median EC_50_ for VOZ compared to *F. solani* SC (*P* < 0.05). A small subset of *F. oxysporum* SC and *F. fujikuroi* SC isolates displayed VOZ EC_50_ values below 1 µg/mL, evident as a pronounced leftward tail in the distribution. In contrast, both *F. oxysporum* SC and *F. fujikuroi* SC exhibited significantly less susceptibility to ITZ, FCZ, and CLZ than *F. solani* SC (*P* < 0.001), as indicated by the shortening of leftward tails in the respective plots (Fig. 2A). Specifically, the complete lack of a leftward tail for *F. oxysporum* SC against POZ suggests uniform poor susceptibility to POZ within the group.

In contrast to clinical azoles, *F. solani* SC displayed a higher median to field azoles, with fewer susceptible strains exhibiting EC_50_ values below 1 µg/mL (Fig. 2B). Consistent with GM values (Table 2), *F. fujikuroi* SC demonstrated significantly higher susceptibility to agricultural azoles, particularly EPO and OXI, compared to the other two species. This difference was statistically significant across all agricultural azoles when compared to *F. solani* SC.

**Fig 3:**
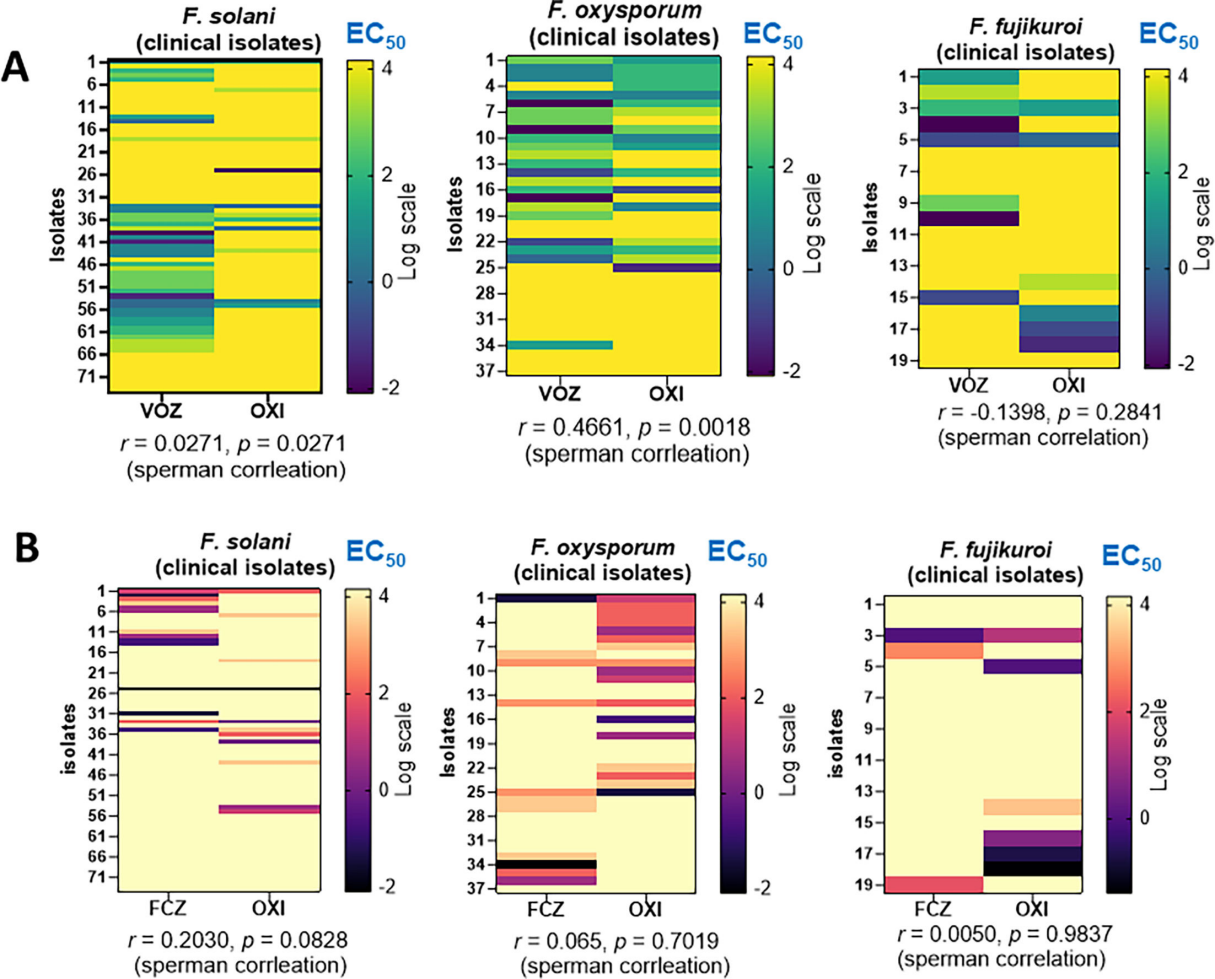
Resistance correlation heatmaps for three *Fusarium* SCs in 130 clinical isolates. (**A**) Correlation between the clinical azole VCZ and the agricultural azole OXI is shown. (**B**) Correlation between FCZ and OXI. The data set includes 74 isolates of *F. solani* SC, 37 isolates of *F. oxysporum* SC, and 19 isolates of *F. fujikuroi* SC. Heatmap color scales represent the log-transformed EC_50_ values. Correlation coefficients were calculated using non-parametric Spearman’s correlation, with *P* ≤ 0.05 considered significant.

To investigate the correlation between clinical and agricultural azole susceptibility among these clinical isolates, we selected VOZ and OXI as representative agents from each group, as they demonstrated greater efficacy compared to other azoles. As shown in Fig. 3A *F.oxysporum* SC exhibited a significant positive correlation between responses to VOZ and OXI (r = 0.4661, *P* = 0.0018). *F. solani* SC showed a weak correlation (*P* = 0.05), while no significant correlation was observed in *F. fujikuroi* SC (r = –0.1398, *P* = 0.2841). In contrast, when FCZ and OXI responses were compared, no significant correlation was detected in any of the *Fusarium* SC groups (Fig. 3B). The elevated resistance to agricultural azoles observed in these clinical isolates reinforces the hypothesis of an environmental origin of resistance in *F. oxysporum* SC and perhaps *F. solani* SC.

### Agricultural *F. oxysporum* isolates exhibited reduced susceptibility to VOZ

To assess whether the observed correlation between VOZ and OXI resistance in clinical *F. oxysporum* SC isolates (Fig. 3A) reflects simultaneous, independent resistance development in both clinical and agricultural settings, we analyzed the full-panel azole susceptibility profiles of 42 environmental *F. oxysporum* isolates, hereafter referred to as “Field Foxy,” which were mostly collected from diseased tomatoes and farm runoff water (37).

**Fig 4:**
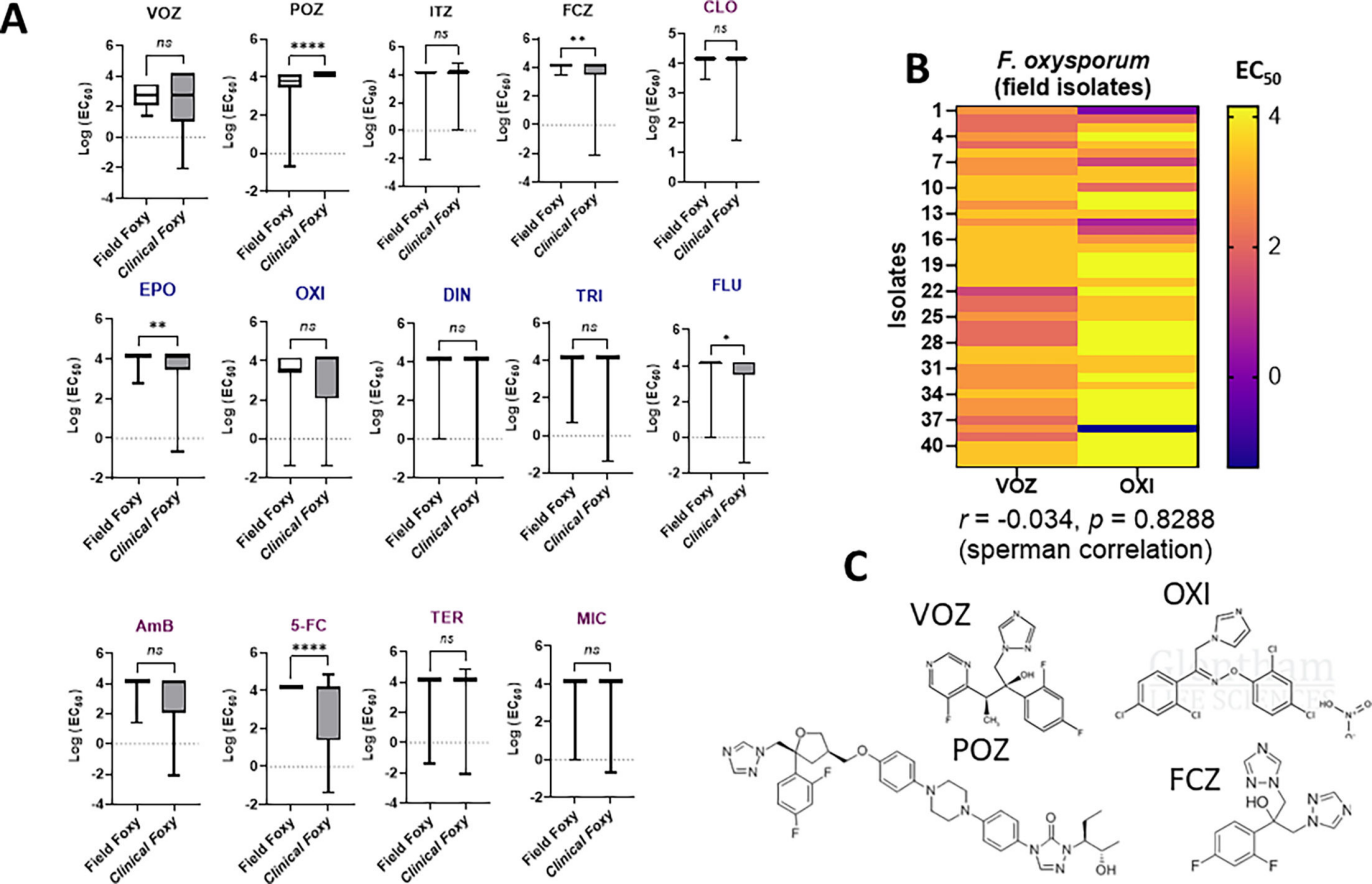
Comparison of antifungal susceptibility profiles between 42 agricultural-associated *F. oxysporum* isolates (Field Foxy) and 37 clinical *F. oxysporum* isolates (Clinical Foxy) against both azole and non-azole antifungals is shown. (**A**) Field Foxy exhibited significantly higher resistance to the agricultural azoles EPO and flutriafol (FLU), but less resistance to the clinical azoles POZ and fluconazole FCZ, compared to Clinical Foxy. Both Field Foxy and Clinical Foxy showed similar resistance patterns for OXI and VOZ. For non-azole antifungals, Field Foxy demonstrated higher resistance to 5-FC. The observed higher resistance to agricultural azoles and 5-FC, alongside susceptibility to POZ and FCZ, suggests possible resistance evolution driven by agricultural fungicide use prior to clinical exposure. EC_50_ medians were compared using one-way ANOVA, followed by the Kruskal–Wallis test. Significance levels: *P < 0.05, **P < 0.01, ***P < 0.001, ****P < 0.0001. (**B**) No significant correlation was found between VOZ and OXI susceptibility in Field Foxy (Spearman’s *P* = 0.83), in contrast to Clinical Foxy, possibly due to higher OXI resistance in field isolates. (**C**) A schematic showing the structural similarity between VOZ and OXI, which may explain their similar resistance profiles. Both share planar aromatic features, while POZ has a bulkier, more hydrophobic structure with extended side chains. FCZ, in contrast, lacks bulky groups.

As expected, compared to clinical *F. oxysporum* isolates (Clinical Foxy), many of the agriculture-associated strains exhibited significantly reduced susceptibility to agricultural azoles, particularly to EPO and FLU (Fig. 4A) and equally reduced susceptibility to OXI. They also showed intrinsic resistance to the clinical azole FCZ and exhibited comparable VOZ susceptibility to the clinical isolates, despite no known prior VOZ exposure. Most field strains displayed intermediate VOZ susceptibility (8–16 µg/mL) (Fig. 4A). Among these field isolates, a positive correlation between VOZ and OXI susceptibility was not observed (Fig. 4B), likely due to their higher resistance to OXI (32–64 µg/mL), in contrast to the significant correlation seen in clinical isolates (Fig. 3A).

Notably, in contrast to the clinical group, the field isolates were more susceptible to POZ (Fig. 4A). Structurally, VOZ and OXI share some spatial similarity due to their relatively compact and planar aromatic scaffolds, which may favor comparable binding interactions with the CYP51 enzyme. By contrast, the smaller and chemically distinct FCZ scaffold is generally associated with weaker CYP51 affinity and limited intrinsic activity against filamentous fungi, whereas POZ is markedly bulkier, featuring a long, hydrophobic side chain with distinct extensions (Fig. 4C). These differences in structural compatibility with CYP51, along with other factors such as genetic mutations, altered gene expression, and variability in efflux activity (27) among strains, likely contribute to the divergent susceptibility patterns observed across FCZ, POZ, and VOZ in both clinical and field isolates. Taken together, these findings suggest that high-level OXI resistance in the agricultural environment may represent one potential contributor to the VOZ resistance observed in clinical *F. oxysporum* strains.

### The susceptibility to non-azole antifungals

Four classes of non-azole clinical antifungals—AmB, 5-FC, TER, and MIF—were included in the resistance trend analysis using the same set of 130 clinical isolates. Unlike clinical azoles, each of these drugs showed a small subset of *Fusarium* isolates with EC_50_ values below 1 µg/mL.

**Fig 5:**
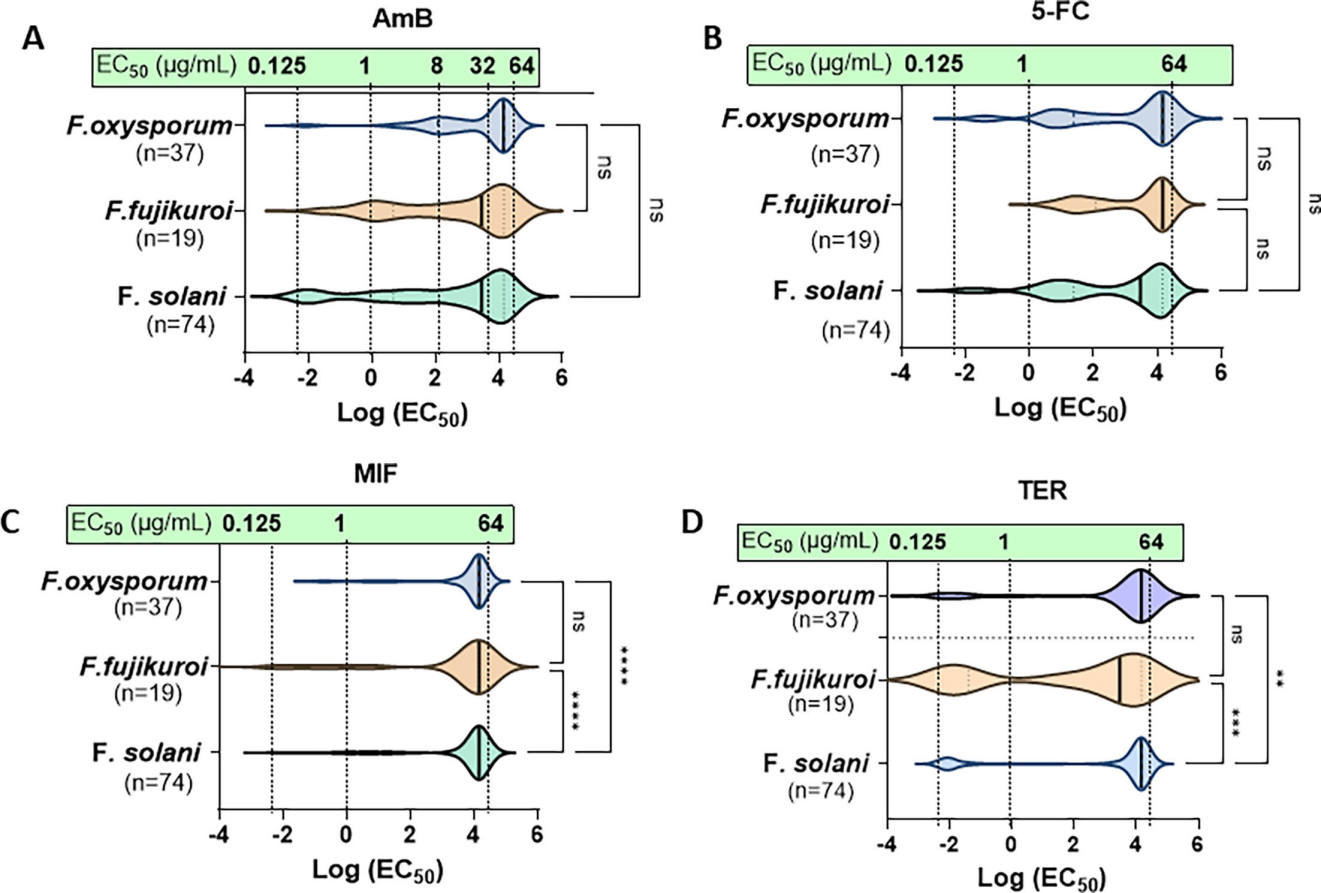
Violin plot of EC_50_ distribution for 130 clinical *Fusarium* isolates against non-azole antifungals, including (**A**) AmB, (**B**) 5-FC, (**C**) MIF, and (**D**) TER. No significant differences in EC_50_ values for AmB and 5-FC were observed among the three SCs (*F. solani* SC, *F. oxysporum* SC, and *F. fujikuroi* SC). However, *F. fujikuroi* showed more susceptibility to TER. The x-axis is presented on a natural logarithm (LN) scale, with corresponding EC_50_ values (µg/mL) overlaid on each graph and connected by a dotted line. Medians between groups were statistically analyzed using one-way ANOVA, followed by the Kruskal-Wallis test. Significance levels are indicated as follows: *P < 0.05, **P < 0.01, ***P < 0.001, and ****P < 0.0001.

For AmB and 5-FC, median EC_50_ values showed no significant difference among the three *Fusarium* SCs (Fig. 5A and B). However, the GM EC₅₀ values for AmB exceeded the established ECVs in all SCs: 10.3 µg/mL for *F. solani* SC, 18.94 µg/mL for *F. oxysporum* SC, and 13.33 µg/mL for *F. fujikuroi* SC, compared to respective ECVs of 8, 8, and 4 µg/mL (37) (Table 1). For 5-FC, although overall susceptibility medians were not significantly different across SCs, *F. fujikuroi* SC exhibited a higher GM (24.79 µg/mL) than *F. solani* SC (14.73 µg/mL) and *F. oxysporum* SC (17.59 µg/mL).

When using these non-azoles against field *F. oxysporum* isolates, we observed their significantly higher reduced susceptibility to 5-FC, with comparable resistance levels of clinical isolates to AmB, TER, and MIF (Fig. 4A). This could result from a broader resistance selection pressure targeting DNA biosynthesis in the agricultural field (38).

### High multiple drug resistance rates to azoles, 5-FC, and AmB among clinical isolates

Given the variable responses of clinical isolates to individual azoles, we defined multiple drug resistance (MDR) categories as follows: R1: resistance to all five clinical azoles (VOZ, POZ, ITZ, FCZ, and CLZ), with EC_50_ values ≥32 µg/mL; R2: resistance to all five agricultural azoles (EPO, OXI, DIN, FLU, and TRI), using the same EC_50_ threshold; R1/R2: cross-drug resistance (CDR) to both clinical and agricultural azoles; R3: resistance to AmB, with EC_50_ ≥32 µg/mL; R4: resistance to 5-FC, with EC_50_ ≥32 µg/mL; R1/R3: MDR to azoles and AmB; R1/R4: MDR between azole and 5-FC; and R1/R3/R4: pan-resistance to azoles, AmB, and 5-FC.

**Fig 6:**
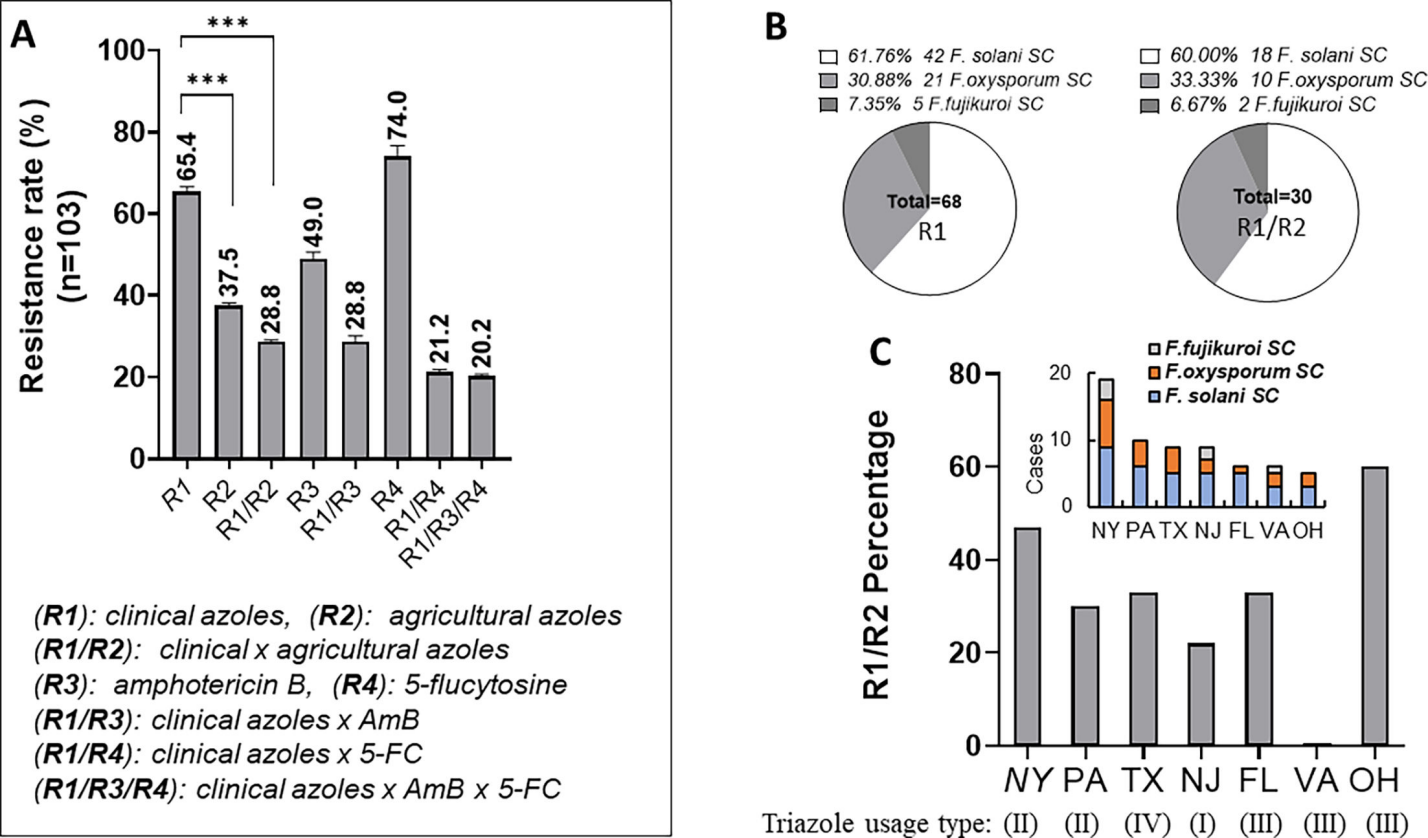
CDR to clinical and agricultural azoles, MDR between azoles and either AmB or 5-FC, and pan-drug resistance (PDR) across three antifungal classes in 103 infected toenail isolates. (**A**) The resistance rates to individual antifungals were 74% for 5-FC (R4), 65.4% for clinical azoles (R1), and 49.0% for AmB (R3). MDR rates (R1/R3 or R1/R4) and PDR rates (R1/R3/R4) were below 30% in this *Fusarium* cohort. (**B**) The overall ratio of species distribution (4:2:1) was consistent across the clinical azole-resistant group R1 (*n* = 68) and the combined azole-resistant group R1/R2 (*n* = 30). (**C**) The distribution of R1/R2 isolates was higher in Ohio (OH), Pennsylvania (PA), and Texas (TX), with no *F. fujikuroi* isolates detected in these states. In contrast, lower R1/R2 prevalence was observed in states where *F. fujikuroi* was present, such as Virginia (VA) and New Jersey (NJ).

Among the 103 *Fusarium* isolates from nail samples (Set C), 28.8% exhibited cross-resistance to both clinical and agricultural azoles (R1/R2, Fig. 6A). This rate was similar to the 27.6% (8/29) observed in Sets A and B. Notably, resistance to clinical azoles alone (R1, 65.4%) was significantly higher than resistance to agricultural azoles alone (R2, 37.5%) with *P* < 0.001, suggesting some clinical origins of the observed azole resistance.

For resistance across drug classes, despite the mono-drug 5-FC resistance rate (74%) being higher than that of AmB (49%), the MDR rate combining clinical azoles with AmB (R1/R3) or 5-FC (R1/R4) is similar, presenting by 28.8 and 21.2%, respectively. Importantly, pan-drug resistant (PDR) to all three (R1/R3/R4) also showed 20.2% among these clinical isolates.

Among the 30 CDR (R1/R2) isolates in Set C (Table 1), the species distribution across the *F. solani*, *F. oxysporum*, and *F. fujikuroi* SCs closely resembled that observed in the R1 group **(**Fig. 6B). However, the overall CDR distribution deviated from the expected 4:2:1 ratio among the three SCs (Fig. 1A), with relatively high proportions of *F. solani* SC and *F. oxysporum* SC and a notably lower proportion of *F. fujikuroi* SC. Similarly, in Sets A and B, *F. solani* SC predominated the R1/R2 isolates, accounting for 68.4% of the group.

Geographic analysis (Fig. 6C) showed that CDR prevalence was highest in Ohio, where *F. fujikuroi* SC was absent, and lower in New Jersey and Virginia, where this SC was present. Notably, three *F. fujikuroi* isolates from Georgia, California, and Alabama were resistant to clinical azoles but remained susceptible to agricultural azoles, despite these states having the highest levels (IV–V) of agricultural triazole usage. Conversely, CDR *F. fujikuroi* isolates were found in New York (level II) and Virginia (level III), both with moderate agricultural azole applications. These findings suggest that CDR is widely prevalent in *F. solani* SC and *F. oxysporum* SC, regardless of clinical or agricultural origin. In contrast, *F. fujikuroi* appears more susceptible to agricultural azoles (Fig. 2B).

## DISCUSSION

This study provides an updated, comprehensive analysis of antifungal susceptibility in 130 clinical *Fusarium* isolates collected across 26 US states. By comparing these isolates with agricultural strains tested against the same antifungal panel, we identified several key patterns. First, the *F. solani* SC and *F. oxysporum* SC predominated among clinical isolates, with a smaller proportion of *F. fujikuroi* SC, at an approximate ratio of 4:2:1. This distribution is consistent with prior reports (39, 40) while offering new geographic insights. Second, we observed a strikingly high level of azole resistance—65.4% of clinical isolates were resistant to VOZ, and resistance to POZ was complete (100%) within the *F. oxysporum* SC. Third, cross-resistance between clinical (VCZ) and agricultural (OXI) azoles was particularly notable in *F. oxysporum* SC and *F. solani* SC, with resistant strains commonly originating from regions with intensive agricultural azole use. In contrast, *F. fujikuroi* SC showed higher susceptibility to agricultural azoles and no consistent cross-resistance, which may explain its lower prevalence in high azole-use states. Finally, resistance extended beyond azoles: 74% of isolates were resistant to 5-FC and 49% to AmB, although rates of MDR were lower (21.2%–28.8%), and pan-resistance was observed in 20.2% of isolates.

Taken together, these findings suggest that environmental azole exposure may contribute to the emergence of clinical *Fusarium* resistance, echoing concerns raised for *Aspergillus fumigatus* (33, 41, 42). However, the higher azole resistance in clinical isolates (R1 >R2 in Fig. 6), coupled with greater agricultural resistance in environmental isolates, also points to the possibility of parallel, independent resistance pathways. *Fusarium*’s adaptation to environmental niches with high oxygen availability and lower temperatures versus host body temperature, biofilm formation, and specific genetic traits may further facilitate both survival in diverse environments and acquisition of azole resistance (17, 27). In *A. fumigatus*, aerosolized azole-resistant spores from the environment have been linked to infections in azole-naïve patients with poor clinical outcomes (43). This raises similar concerns that resistant *Fusarium* strains could likewise originate in agricultural environments before establishing in clinical settings.

The compound-specific patterns of cross-resistance also merit consideration. Structural similarities between VCZ and OXI, both compact aromatic molecules, may underlie their shared resistance profiles in *F. oxysporum* SC (and to a lesser extent *F. solani* SC). In contrast, the lack of correlation between FCZ and OXI resistance, as well as the discordant POZ susceptibility patterns between clinical and agricultural *F. oxysporum* isolates, indicates that cross-resistance is not universal across azole subclasses. Given the widespread use of OXI in agriculture, chronic exposure has likely selected for resistant *F. oxysporum* and *F. solani* populations. The comparatively higher susceptibility of *F. fujikuroi* SC to agricultural azoles, together with its resistance to VCZ in clinical settings, suggests hospital-based selective pressures may be more relevant for this group.

Therapeutically, our data emphasize the limited options for fusariosis management. Resistance to 5-FC and AmB was common, though the narrow EC_50_/EC_90_ range for 5-FC and its relatively better performance against *F. oxysporum* (Fig. 4A) suggests that DNA synthesis inhibition remains a potential antifungal avenue (38). Notably, the benzimidazole fungicide carbendazim (CBZ), having been widely used in agriculture, also interferes with fungal DNA synthesis during mitosis, potentially contributing to 5-FC resistance mechanisms. While resistance to single agents was high, the lower rates of MDR and pan-resistance indicate that combination therapy—particularly azoles with AmB or 5-FC—may retain some efficacy. TER, either alone (44) or in combination with AmB (45), has demonstrated preferential activity against *Fusarium*, highlighting another possible avenue for treatment.

It is important to note that toenail isolates comprised the majority of clinical samples, reflecting the chronicity of onychomycosis and potential selection from either environmental reservoirs or prolonged azole therapy. Azoles are typically prescribed for 3–4 months to treat dermatophyte infections (27, 46), and such regimens may inadvertently select for resistant *Fusarium* strains. Clinically, second-generation triazoles such as VCZ and POZ remain first-line treatments for fusariosis; however, our finding of widespread VCZ resistance and poor POZ responsiveness underscores the urgency of the therapeutic challenge. Indeed, the lack of POZ efficacy *in vitro* is consistent with clinical reports of breakthrough infections during prophylaxis (26).

This study has several limitations. First, we did not directly link antifungal susceptibility profiles to clinical outcomes, which restricts the interpretation of therapeutic impact. Second, although we provide a comprehensive analysis of phenotypic resistance, the absence of molecular data, such as target gene mutations or efflux pump expression (currently under investigation), may constrain the interpretation of resistance mechanisms. Third, isolates were collected through non-stratified random sampling across 26 states, which introduces geographic and temporal biases and limits global extrapolation. Furthermore, because *F. solani* and *F. oxysporum* are major plant pathogens with extensive agricultural exposure to azoles, broader and more stratified environmental sampling will be essential to fully understand the ecological drivers of resistance.

In summary, this study demonstrates the high prevalence of azole resistance among U.S. clinical *Fusarium* isolates and provides evidence for potential environmental contributions. The findings highlight the urgent need for novel antifungal agents, optimized combination regimens, and integrated approaches that address both clinical and environmental sources of resistance. Future work should prioritize elucidating molecular mechanisms of cross-resistance and developing targeted therapeutic strategies for these resilient pathogens.

### Conclusion

This study highlights the high resistance rates to both clinical and agricultural azoles among *Fusarium* species, with *F. oxysporum* SC and *F. solani* SC showing strong links to environmental azole exposure. In contrast, *F. fujikuroi* SC resistance appears more likely to arise in clinical settings. While mono-therapy options for *Fusarium* infections remain limited, the lower rates of MDR and PDR across antifungal classes suggest that combination therapies may still hold promise. Ongoing surveillance and integrated treatment strategies are essential to address the growing challenge of antifungal resistance in *Fusarium* infections.

## MATERIALS AND METHODS

### Strains and growth conditions

A total of 174 *Fusarium* isolates were used in this study, originating from four sources, designated as Set A, Set B, Set C, and Set D, as shown in Table 1. The first three sets consist of clinical isolates, while Set D comprises field isolates.

Set A includes 17 *Fusarium solani* isolates (A1–A17) provided by the CDC Hurst Lab. Set B consists of 12 isolates (B1–B12) obtained from Dr. Nathan P. Wiederhold at the University of Texas Health Science Center at San Antonio, collected from different anatomical sites across multiple states; however, the collection periods for Sets A and B are unknown. Set C includes 103 strains collected from toenail infections through a diagnostic service company in Georgia, which received samples nationwide between April and June 2024. All clinical isolates were identified using MALDI-TOF as part of routine fungal pathogen diagnostics.

Set D primarily consists of *Fusarium oxysporum* f. sp. *lycopersici* (FOL) isolates originally obtained from tomato cultivars in North Carolina between 2017 and 2018 (37), which have undergone molecular identification. Additional isolates were collected in March 2024 from Maryland soil samples.

The primary growth medium used for *Fusarium* isolates in this study was potato dextrose agar (PDA; MP Biomedicals, Irvine, CA, USA). For long-term storage, spores and mycelial blocks were preserved in PDA supplemented with 15% glycerol at −80°C. New cultures were established by inoculating a 0.5 cm² mycelial block in the center of a PDA plate and incubating it at 30°C for 6 days. To prepare a spore suspension, the mold growth on PDA was scraped with a sterilized blade, and spores were harvested in 5 mL of 1% Tween 20 (polysorbate 20 in Tris-buffered saline, pH 7.4) (47). The suspension was allowed to settle briefly to let large mycelial fragments sink. The upper portion, containing spores and short hyphal cells, was quickly transferred to a new 15 mL-BD tube as the fungal stock. Spore concentrations were determined using a white blood cell (WBC) hemocytometer.

### Testing azoles and other compounds

A total of 14 antifungal compounds were selected to evaluate the drug susceptibility of our *Fusarium* collection, including TER, AmB, MIF, 5-FC, and 10 azoles. All compounds were stored at −20°C

Among the 10 azoles, five are clinically used: fluconazole (FCZ), itraconazole (ITZ), voriconazole (VOZ), posaconazole (POZ), and the topical azole clotrimazole (CLZ), abbreviated using the first two letters followed by “Z.” The remaining five are agricultural azoles: epoxiconazole (EPO), triflumizole (TRI), flutriafol (FLU), diniconazole (DIN), and oxiconazole (OXI), abbreviated with the first three letters to distinguish them from clinical azoles in the following analysis.

FCZ was obtained from LKT Laboratory, Inc. (Cat. F4682); ITZ from Spectrum Chemical MFG. Corp. (Cat. I2202, Gardena, CA); VOZ from Pfizer, Inc. (Cat. UK-109496); and POZ from AmBeed (Cat. A133379). MIF was sourced from Astellas Pharma US, Inc. (Deerfield, IL), and TER from Cayman Chemical Co. (Cat. 10011619). Stock solutions for all compounds were prepared at 5–10 mg/mL in DMSO and stored at −20°C.

### MIC assay in RPMI-1640 medium

MIC assays were performed following the guidance from CLSI (the Clinical and Laboratory Standards Institute, M-38) (48). RPMI-1640 (Sigma-Aldrich) containing 2 mM L-glutamine, 10 mM HEPES, and 1 mM sodium pyruvate, then supplemented with 2% dextrose (D-glucose anhydrous; Sigma-Aldrich) and MOPS (3-(N-morpholino propanesulfonic acid; Fisher Scientific, Waltham, MA, USA) buffered to pH 7.0.

Each compound was prepared in a 96-well plate (Nunclon Delta Surface, Thermo Scientific) by serial twofold dilution in RPMI-1640, yielding final concentrations ranging from 128 to 0.125 µg/mL (#1 to #11 columns). Conidial suspensions were prepared from spores and short hyphal fragments collected in 0.1% Tween 20 saline, then adjusted to 1 × 10^4^ conidia/mL in RPMI-1640 broth. A 0.1 mL aliquot of the fungal suspension was added to each well containing the compound dilution series. To distinguish the MIC_50_ and MIC_90_, which describe population-level inhibition in this study, we used EC_50_ and EC_90_ to represent the lowest effective concentration required to achieve 50% and 90% inhibition for a single strain. Cultures were incubated at 30°C for 48 hours, with drug-free wells (column #12) serving as controls. The MIC was determined by measuring the optical density (OD) at 590 nm using a TRIAD Series Multimode Detector (Dynex Technologies, USA) (49).

### Determination of EC_50_ values for growth inhibition with reported MIC ECVs

Currently, there are no established clinical breakpoints for antifungals against Fusarium species. To better present resistance trends and interpret MIC data, we applied ECVs to define resistance thresholds, differentiating wild-type (WT) from non-WT (NWT) fungal populations. ECVs represent the highest MIC expected for organisms without detectable resistance mechanisms and provide a standardized approach to identify isolates with acquired or mutational resistance, particularly when conventional breakpoints are not available.

We followed the ECVs reported by Espinel-Ingroff et al. (36), which are as follows: AmB at 4 µg/mL for *Fusarium fujikuroi* SC and 8 µg/mL for *Fusarium oxysporum* SC and *Fusarium solani* SC; POZ at 2 µg/mL, 8 µg/mL, and 32 µg/mL for *F. fujikuroi* SC, *F. oxysporum* SC, and *F. solani* SC, respectively; VOZ at 4 µg/mL, 16 µg/mL, and 32 µg/mL for *F. fujikuroi* SC, *F. oxysporum* SC, and *F. solani* SC, respectively; and ITZ at 32 µg/mL for all tested strains. For antifungals without established ECVs, including agricultural azoles and non-azoles (except AmB), we used 32 µg/mL as the cutoff threshold.

To better present the individual antifungal susceptibility profiles in these clinical isolates, the EC_50_ values (growth inhibition for individual strains) for clinical azoles (VOZ, POZ, ITZ, and FCZ) and agricultural azoles (EPO, TRI, FLU, DIN, and OXI) were utilized to calculate the GM, population-level MIC_50_, and MIC_90_ values. The population-level MIC_50_ and MIC_90_ values represent the drug concentrations required to inhibit the growth of 50% or 90% of tested strains, respectively.

### Statistical analysis

All EC_50_ data were analyzed using GraphPad Prism 5. To compare drug susceptibility trends among the three *Fusarium* SC groups, violin plots displaying median values were generated. Differences among groups were assessed using one-way analysis of variance (ANOVA) followed by Dunnett’s post hoc test. For non-normally distributed data, the Kruskal-Wallis test was used instead. Pairwise comparisons between two groups were conducted using an unpaired t-test or the Mann-Whitney U test when normality assumptions were not met. The correlation between resistance to clinical and field azoles in each *Fusarium* SC group, or between *Fusarium* isolates from clinical and environmental sources, was evaluated using non-parametric Spearman correlation analysis with a two-tailed approach to obtain correlation coefficients. All statistical significance was set at *P* ≤ 0.05.
